# Opening the Black Box of Daily Life in Nonsuicidal Self-injury Research: With Great Opportunity Comes Great Responsibility

**DOI:** 10.2196/30915

**Published:** 2021-11-19

**Authors:** Glenn Kiekens, Kealagh Robinson, Ruth Tatnell, Olivia J Kirtley

**Affiliations:** 1 Faculty of Psychology and Educational Sciences, Clinical Psychology KU Leuven Leuven Belgium; 2 Department of Neurosciences, Center for Contextual Psychiatry KU Leuven Leuven Belgium; 3 School of Psychology Te Herenga Waka-Victoria University of Wellington Wellington New Zealand; 4 Faculty of Health, School of Psychology Deakin University Melbourne Australia

**Keywords:** real-time monitoring, nonsuicidal self-injury, NSSI, experience sampling, ecological momentary assessment, digital psychiatry

## Abstract

Although nonsuicidal self-injury (NSSI)—deliberate damaging of body tissue without suicidal intent—is a behavior that occurs in interaction with real-world contexts, studying NSSI in the natural environment has historically been impossible. Recent advances in real-time monitoring technologies have revolutionized our ability to do exactly that, providing myriad research and clinical practice opportunities. In this viewpoint paper, we review new research pathways to improve our ability to understand, predict, and prevent NSSI, and provide critical perspectives on the responsibilities inherent to conducting real-time monitoring studies on NSSI. Real-time monitoring brings unique opportunities to advance scientific understanding about (1) the dynamic course of NSSI, (2) the real-time predictors thereof and ability to detect acute risk, (3) the ecological validity of theoretical models, (4) the functional mechanisms and outcomes of NSSI, and (5) the promotion of person-centered care and novel technology-based interventions. By considering the opportunities of real-time monitoring research in the context of the accompanying responsibilities (eg, inclusive recruitment, sound and transparent research practices, participant safety and engagement, measurement reactivity, researcher well-being and training), we provide novel insights and resources to open the black box of daily life in the next decade(s) of NSSI research.

## Introduction

Nonsuicidal self-injury (NSSI), defined as the direct and deliberate damage of one’s body tissue without suicidal intent (eg, cutting and hitting oneself) [1], is a behavior seemingly at odds with the principles of minimizing pain and maximizing pleasure, which guide most human behaviors. One in 5 people engage in NSSI at least once before the age of 25 years [2,3], and doing so increases their risk for future suicidal thoughts and behaviors and mental health conditions [4,5] and other adverse developmental outcomes [6-8]. Unfortunately, few individuals access support for their NSSI [9], with many young people who self-injure not finding their way to treatment [10]. Together, these findings highlight NSSI as behavior that warrants greater awareness and a better understanding—a viewpoint that the American Psychiatric Association formally emphasized by including NSSI as a “condition requiring further study” in the fifth edition of the Diagnostic and Statistical Manual of Mental Disorders [11].

Taking stock of the research published in the past decade reveals substantial advances in our understanding of the epidemiology, phenomenology, and developmental course of NSSI [2,3,10,12]. Longitudinal cohort studies have substantially advanced knowledge regarding intrapersonal and interpersonal risk and protective factors that clarify who is at the highest risk for developing [13,14] and continuing NSSI behavior during adolescence and emerging adulthood [10,15,16]. Unfortunately, our understanding of when young people are at risk of NSSI in everyday life has not progressed similarly. We see 3 main reasons hindering this knowledge progression. First, it is an ecological fallacy to believe that a nomothetic approach that provides between-group knowledge about who is relatively at high risk throughout adolescence and emerging adulthood can be translated to the here and now at the individual level [17,18]. Indeed, knowing that someone is developmentally at risk to engage in NSSI (eg, due to a history of victimization) [19] tells us little about *when* that person is most likely to self-injure in everyday life. Second, nearly all longitudinal research studies used observation windows from months to years to clarify developmental risk [20]. However, retrospectively aggregating data over months to years (eg, *Have you self-injured since last year?*) lacks the temporal precision to detect individual risk within minutes-to-hours. Third, and perhaps most importantly, researchers have historically been constrained by practical restrictions that rendered frequent assessments of NSSI in individuals’ daily life virtually impossible. Nevertheless, in a new era of precision medicine, if we are to enable individualized intervention *when* and *where* it is most needed, then research needs to take an idiographic approach in which risk stratification repeatedly occurs in the natural environment with individuals serving as their own control [18].

## Out of the Laboratory and Into Everyday Life

Recent advances in digital technology now make it possible for researchers to take such an idiographic approach, shifting research from the laboratory into the everyday environment where NSSI thoughts, urges, and behaviors occur. Real-time monitoring (also called experience sampling or ecological momentary assessment) is a structured self-report diary technique in which individuals provide information on their situational context, feelings, thoughts, and behavioral patterns in the flow of daily life [21,22]. Self-report questionnaires are completed *multiple times* throughout the day for several days or weeks. Participants are prompted to fill in questionnaires either during predetermined intervals of time (eg, every 2 hours, interval-contingent sampling) at random unpredictable moments (ie, signal-contingent sampling) or following an event of interest (ie, event-contingent sampling) [23]. Daily diaries are a particular case of interval-contingent sampling in which assessments occur only once and typically at the end of each day. Real-time monitoring methods are not a new methodology [24], with roots in ecological psychology, which argues that behavior can only be understood when investigated in the context in which it occurs [25]. Although initial real-time monitoring studies of NSSI relied upon pagers and personal digital assistants [26,27], the ubiquity of mobile smartphones in today’s society [28] has made it practically feasible for the broader research community to study NSSI and its contextual determinants in everyday life. The increased practicality of real-time monitoring methods offers a promising avenue to answer critical questions and engage researchers and clinicians in collaborative discussions. However, real-time monitoring methods, which focus on NSSI, also present significant ethical and practical challenges.

Given that real-time monitoring research is burgeoning [29-31], it is timely to consider the valuable new directions in which the field could be heading when studying NSSI outside the laboratory, in everyday life. In 2019, the International Society for the Study of Self-Injury established a *Consortium for Research on Self-Injury in Everyday Life* to help build expertise and capacity in a rapidly growing field [32]. In this Viewpoint paper, arising from the work of the consortium, we (1) review new research pathways that use real-time monitoring methods to improve our ability to understand, predict, and prevent NSSI, and (2) provide critical perspectives on the responsibilities inherent to conducting real-time monitoring studies on NSSI. In doing so, we identified crucial open questions that require further investigation and offer guidance and concrete recommendations for future studies.

## Opening the Black Box of Daily Life Brings Exciting New Opportunities for Science and Practice

In this section, we outline 5 key opportunities that real-time monitoring provides for advancing our ability to understand, predict, and prevent NSSI thoughts, urges, and behaviors in the lives of those at risk (Textbox 1).

**Five key opportunities of real-time monitoring.**

New opportunities created by opening the black box of daily life in nonsuicidal self-injury research:

- Better understanding of the short-term course of nonsuicidal self-injury thoughts, urges, and behavior through direct observation and precise measurement.
- Advance knowledge of individual-level predictors of nonsuicidal self-injury thoughts, urges, and behavior and the ability to accurately detect idiographic risk.
- Test existing theories and develop new models that bridge the idiographic and nomothetic divide and explain who is at risk and when.
- Generate insights into the functional mechanisms and relationship of dynamic patterns with day-to-day and meaningful longer term developmental changes and outcomes.
- Promote person-centered care and deployment of personalized prevention and novel digital interventions.

### Opportunity 1: A Better Understanding of the Short-term Course of NSSI Thoughts, Urges, and Behavior Through Direct Observation and Precise Measurement

Real-time monitoring enables rigorous descriptive research about the course of NSSI thoughts, urges, and behaviors. Initial work has demonstrated that NSSI thoughts frequently occur among individuals who self-injure but are usually short-lived and are of moderate intensity [27,33,34]. The propensity to experience intense and persistent NSSI thoughts has been found to increase throughout the day [34], with thought intensity fluctuating considerably from hour to hour for some individuals [35]. However, future work is needed to replicate these findings and many questions remain, including dynamics over even shorter timescales (ie, within seconds/minutes), whether different thought profiles can be identified in terms of intensity, duration, controllability, and persistence, and the degree to which changes in dynamic thought patterns relate to urges and behaviors.

Different qualitative aspects of NSSI thoughts and urges may combine to increase risk, such that the likelihood of NSSI behavior may increase in situations characterized by more intense persistent thoughts [33]. In this respect, real-time monitoring offers the opportunity to capture a fast-moving thought-to-action process through precise measurement in real time. Research suggests that it typically takes people 1-30 minutes to transition from NSSI thoughts to behavior [27,33], meaning that in most instances, there is a brief window of opportunity to intervene and interrupt the transition from thoughts to behavioral action. Better characterization of the thought profiles and behavioral patterns of NSSI as well as the extent to which these can change both within and across individuals are an essential first step in identifying individual-level predictors for risk screening and preventive intervention.

### Opportunity 2: Advancing Knowledge of Individual-Level Predictors of NSSI Thoughts, Urges, and Behavior, and the Ability to Accurately Detect Idiographic Risk

Daily life research provides a contextualized understanding of the momentary factors that explain variability in the short-term course of NSSI. Using real-time monitoring, researchers can study theoretically relevant situational, emotional, and cognitive factors to advance knowledge of individual-level predictors for developing NSSI thoughts and urges, and for making the transition to behavior. Initial findings suggest that the likelihood of these outcomes may increase when people are alone [27], after negative social appraisals and perceived conflict [36,37], or following increased negative and decreased positive affect [35,38]. Studies have also observed an increased risk of NSSI thoughts, urges, and behavior in the presence of high self-criticism and negative repetitive thinking [39,40], or low momentary self-efficacy to resist NSSI [35]. Despite this knowledge, future research is needed to clarify the relative importance of these situational, emotional, and cognitive factors at each stage of the NSSI process and their specificity in predicting NSSI compared to co-occurring behaviors (eg, eating disorder behaviors, suicidal thoughts) [27,41]. Worth mentioning in this context is that real-time monitoring also provides a unique opportunity to clarify the relationship with these comorbid behaviors in daily life [42], thereby offering meaningful information to further diagnostic understanding of NSSI. Finally, the timescale in which factors exert an effect and how their interplay can be understood mathematically (ie, linear or nonlinear effects) warrants further clarification.

Building upon empirically derived answers to these critical questions, the next fundamental step is developing risk prediction models that can accurately detect *when* someone is at imminent risk for engaging in NSSI. Using each individual’s longitudinal data, researchers can select and combine risk and protective factors to create risk stratification indices of NSSI thoughts, urges, and behavior in the natural environment for a particular person (eg, in the case of smoking behavior) [43]. Statistical classification approaches (also known as machine learning) and validation techniques can be employed to identify the most suitable person-specific combination of risk factors [44,45]. However, 2 caveats should be acknowledged for future research in this area. First, although idiographic risk prediction models will scale up the ability to identify individuals at acute risk for NSSI thoughts, urges, and behavior in daily life, making better use of mobile technologies’ growing capacities will be pivotal to ensure that individuals identified as at-risk are not left without the necessary support (*see opportunity 5*). Second, because real-time monitoring for prolonged periods becomes burdensome, it will be crucial to capitalize on the ever growing technological capacities and explore the utility and integration of passively collected information in these models [46]. For example, smartphones continuously track a wealth of “in the moment” information (eg, call or SMS logs, location recording), and initial investigation supports the feasibility of using wearables to measure psychophysiology among high-risk adolescents [47]. Importantly, these 2 caveats illustrate that real-time monitoring of NSSI thoughts and behaviors also brings considerable ethical, legal, and practical challenges regarding inclusivity, informed consent, and participant safety and burden (*see responsibilities section for a discussion of these challenges*).

### Opportunity 3: Test Existing Theories and Develop New Models That Bridge the Idiographic and Nomothetic Divide and Explain Who Is at Risk and When

Real-time monitoring provides researchers with the opportunity to put existing theories to the test in daily life. Contemporary theories of NSSI posit that the joint influence of social, affective, and cognitive vulnerabilities cause risk for NSSI via idiographic microprocesses that play out in the realm of ordinary life. However, these psychological processes are typically evaluated using cross-sectional and traditional longitudinal surveys—designs that do not have the necessary temporal granularity or ecological validity to reliably assess these theories’ dynamic real-life components. Real-time monitoring overcomes this limitation. For instance, Hughes and colleagues [40] observed that momentary negative affect and repetitive negative thinking synergistically predict NSSI in daily life, thereby providing evidence for the Emotional Cascade Model [48]. A limitation of this model is that it does not address *why* someone chooses to engage in NSSI instead of other dysregulated behaviors. In this respect, the Benefits and Barriers Model argues for the unique role of self-criticism in developing NSSI [49], whereas the Cognitive-Emotional Model argues for an expanded role of NSSI-specific cognitions [50]. Consistent with the Cognitive-Emotional Model, cross-sectional evidence suggests that behavior-specific beliefs (eg, self-efficacy to resist NSSI) explain why individuals use NSSI instead of risky alcohol use or disordered eating when distressed [51]. Investigations of these models in daily life are currently ongoing [35,39,52]. Emerging evidence, for instance, suggests that momentary belief in one’s ability to resist NSSI is a robust short-term predictor of NSSI behavior among young adults in daily life [35]. However, more work is required to replicate and extend initial findings, including whether behavior-specific beliefs can explain engagement across different behaviors—NSSI and non-NSSI—for everyone.

Notably, existing theories of NSSI do not explicitly differentiate nomothetic and idiographic risk processes, thereby implicitly assuming that what causes risk is the same across individuals. Nevertheless, we can expect that variation in risk processes will be the rule rather than the exception [17,53]. As in most psychology areas [54], existing models are verbal theories, which formulate a narrative of how NSSI behavior manifests rather than translating the theory’s tenets and assumptions into a formal model using mathematical notations. Emerging work underscores the need for novel models in psychology to make formal predictions [54-56], which would allow researchers to precisely estimate what a theory predicts at different measurement levels in computational models and compare this with real-world data. Real-time monitoring can facilitate the generation of theoretical models that conceptualize NSSI as a complex system of contextualized dynamic processes. When formalized, dynamic and contextually informed theories could predict concretely and precisely *when* NSSI thoughts, urges, and behaviors are likely to occur and for *whom*. Such theory construction would progress understanding of factors that increase/decrease the risk for everyone, a subgroup of individuals, or a specific individual [57], and help overcome the research practice gap by allowing practitioners to consider what causes risk for an individual while still enabling the scalability and generalizability of these predictions to be evaluated [58].

### Opportunity 4: Insight Into the Functional Mechanisms, Day-to-day Outcomes, and Relationship of Dynamic Patterns With Meaningful Long-term Developmental Changes and Outcomes

By providing the opportunity to track the dynamic processes in the moments that lead up to and follow self-injurious behavior, real-time monitoring allows investigation of the functional mechanisms that maintain NSSI in daily life. According to the Four-Function Model [59], NSSI may be used to mitigate negative or unwanted thoughts and feelings (ie, intrapersonal negative reinforcement), to generate emotion as a form of stimulation (ie, intrapersonal positive reinforcement), to escape from uncomfortable social situations (ie, interpersonal negative reinforcement), or to seek support from others (ie, interpersonal positive reinforcement). Although the Four-Function Model has received considerable empirical support in cross-sectional studies [12,60], longitudinal measurement in real-life is needed to model the temporal contingencies of interpersonal and intrapersonal processes. A recent review of daily life studies of NSSI revealed the most evidence for intrapersonal negative reinforcement but also found substantial inconsistencies [30]. For example, although some studies observed an increase in negative affect before and a decrease following NSSI behavior [61], others failed to replicate this pattern [62], and some even found increased, rather than decreased, negative affect following NSSI behavior [63]. Although investigations of the other reinforcement processes are scarce, findings were also mixed [30]. An important recommendation for future work is to consider timeframes more carefully. Real-time monitoring studies that add brief follow-up surveys to their protocol when people report momentary NSSI thoughts and urges provide a unique opportunity to unravel contingencies that unfold across shorter (ie, seconds, minutes) and longer (eg, hours, days) time intervals [64].

Apart from providing insight into the functional mechanisms, such studies would also clarify the psychosocial outcomes of NSSI in daily life. For example, engagement in NSSI may lead to interpersonal conflict as well as increased social support [65-67], feelings of shame [68], and experiencing stigma (especially when scars are visible) [69,70], which may, in turn, increase social withdrawal and the likelihood of future NSSI. Hence, much could be learned from future investigations that adopt a transactional framework in which NSSI outcomes and psychosocial experiences might influence each other reciprocally in daily life. Incorporating real-time monitoring within prospective cohort studies (ie, measurement burst designs) [71] can uniquely inform how short-term patterns relate to long-term developmental change and outcomes. Although already employed in depression and substance use research [72,73], these measurement burst designs are currently an untapped resource for NSSI research. For instance, the degree to which NSSI thoughts are self-sustaining in daily life (ie, auto-correlation) could signal a more challenging recovery process [74] or help explain why some individuals (eg, those with depression) are at risk of a more chronic NSSI course [75]. Given the relationship between NSSI and suicidal thoughts and behaviors throughout development [4,76], a critical question is clarifying whether a dynamic blueprint of NSSI can help gauge the future risk of suicidal forms of self-injury. Providing greater clarity regarding potential day-to-day and long-term developmental outcomes would aid scientific understanding and provide valuable information for prevention efforts and clinical risk assessment.

### Opportunity 5: Promotion of Person-Centered Care, Personalized Prevention, and Novel Technology-Based Interventions

Over the last decades, mental health care has gradually shifted from hospital-based to community-based care and changed focus from symptom reduction to patient-defined recovery [21]. Through repeated observation of emotions, thoughts, symptoms, NSSI outcomes, and contextual determinants thereof in patients’ lives, real-time monitoring can help respond to the call for more person‑centered care in the treatment of NSSI [74]. For instance, through easy to interpret visualizations of real-time monitoring data, information on individual functioning and patient-defined outcomes can be fed back into the therapy room. This way, real-time monitoring could facilitate psychoeducation about relevant processes—that patients may otherwise be unaware of—and give clinicians and patients a valuable tool to monitor and tailor treatment according to patients’ dynamic therapy needs. For an example of such a real-time monitoring tool, see the KU Leuven m-Path app and platform [77]. However, to enable the use of real-time monitoring as a therapeutic tool in the treatment of NSSI, pilot studies are required to address the barriers (eg, burden and fear of reactivity) [78] and requirements for successful implementation (eg, availability of an accessible and reliable platform) [79] of real-time monitoring as a blended care tool. Building upon this, randomized controlled trials are needed to determine how, when (eg, unguided in the moment or guided during a clinical session), and which type of feedback (eg, overall functioning, activities, social interactions, or NSSI-specific triggers and risk processes) should be offered. Codeveloping answers to these open questions with all stakeholders involved (ie, people with lived experience, researchers, clinicians, software developers) represents a critical step to harness the potential of real-time monitoring for NSSI treatment.

Finally, real-time monitoring provides scientist-practitioners the opportunity not only to observe but also to deliver support in people’s everyday lives, taking mental health care beyond the clinical setting and into daily life. Ecological momentary interventions (EMIs) are delivered in real time through a smartphone app or a wearable (eg, smartwatch) and can be offered as a self-help mobile health intervention or to augment and extend the reach of existing treatments [21,80]. Initial findings indicate the acceptability and potential of EMIs and mobile apps that target NSSI [81-83], but this remains a largely underexplored area of research. A sophisticated EMI that currently shows promising results in mental health research is just-in-time adaptive interventions (JITAIs) [84], which helps people resist the urge to self-injure when needed most in daily life. JITAIs tailor interventions to the risk status (eg, low, medium, high) and the receptivity of the people within the environmental context, thereby enabling timely and contextually informed interventions for behaviors that are highly dynamic [85]. Give these possibilities, the use of JITAIs is already emerging in suicide research [86], with similar efforts needed to develop, evaluate, and integrate these new treatment methods into a stepped care model for NSSI. 

## Summary of Opportunities

Opening the black box of daily life in NSSI research has considerable potential to advance scientific understanding about (1) the short-term course of NSSI thoughts, urges, and behavior; (2) the individual-level predictors thereof and ability to accurately detect imminent risk; (3) the ecological validity of theoretical models and the possibility to explain *when* NSSI thoughts and behaviors are most likely to occur and *for whom;* (4) the functional mechanisms of NSSI and relationship of dynamic patterns with day-to-day and meaningful long-term change and outcomes; and (5) the implementation of real-time monitoring to prevent key NSSI outcomes and support individuals in distress when they need it the most. However, studying NSSI “in the wild” outside a controlled laboratory environment also presents unique challenges for which there are no established gold standard solutions.

## With Great Opportunity Comes Great Responsibility

In the following section, we outline vital responsibilities when planning and carrying out real-time monitoring research of NSSI thoughts and behaviors (Textbox 2). Although some considerations are universally applicable to real-time monitoring research, here we focus on seven issues that have particular relevance in the context of NSSI and point to open questions in these domains for future research.

**Seven issues that have particular relevance in the context of nonsuicidal self-injury (NSSI).**

Ethical and practical considerations when opening the black box of daily life in NSSI research:

- Recruitment should be inclusive from study inception to completion and actively include more vulnerable individuals, with representatives from any vulnerable group at every stage.
- The informed consent process should be fully transparent regarding the study demands, the safety protocol, whether data will be passively collected, reimbursement, researchers’ responsibility to respond to risk, and potential implications of this responsibility.
- A proper safety protocol should be developed with all stakeholders that matches participants’ needs (especially in the event of suicide risk), but that does not inadvertently defeat the study’s observational purpose.
- Although there is no reason to expect that repeated questioning in everyday life will lead to measurement reactivity in nonsuicidal self-injury outcomes, researchers are responsible for evaluating whether this holds for all participants in their study.
- Study designs must be carefully balanced to appropriately answer the research question(s) while not unnecessarily burdening participants. Sufficient resources should be allocated to pilot all aspects of the protocol. Researchers are encouraged to preregister their protocol and be aware of the relevant privacy laws in their home country before commencing data collection.
- Participants should be recognized as valued contributors to the research and receive financial incentives and information about the overall findings. Where feasible, participants should receive feedback on their own data.
- Research staff should receive good quality training in responding to risk and continued supervision and mentoring. A lone researcher should never be the only person responsible for participants’ safety.

### Responsibility 1: Recruitment and Inclusivity

When the goal is to understand the dynamic course of NSSI, sample diversity—without becoming tokenistic—should be prioritized to safeguard against falsely generalizing from one individual’s (or a subgroup’s) experience to the entire population. Therefore, we recommend actively engaging with members of more vulnerable groups, where the risk of NSSI and suicide may be higher than that in the general population (eg, LGBTQIA+ [lesbian, gay, bisexual, transgender, queer, intersex, asexual], Black, Indigenous, and other people of color, people facing homelessness) [87,88], and utilizing their input on how best to approach the research. Inclusive research is always essential [89], but especially when dealing with sensitive topics such as NSSI. For instance, working with people from different cultural and linguistic backgrounds requires flexibility in the way themes such as NSSI, suicide, and death are considered and discussed owing to differences in cultural norms and language use [90,91]. Some people may also not have access to a smartphone with a 4G connection or might share 1 smartphone in a household or family, conferring additional privacy concerns [92,93]. Therefore, researchers might aim for a budget that allows devices or data bundles to be provided to participants who need them, rather than excluding them. Importantly, however, if the real-time monitoring protocol involves deploying EMIs, researchers should be aware that participants may have come to rely upon the device and the EMI during the study period and that withdrawing these at the end of the study may leave participants without crucial support. Flexible compensation schedules, in which participants can choose to keep the smartphone as compensation for their participation, may be one solution. If practically unfeasible—either because of logistical constraints on the researchers or because the EMI requires a mobile data plan that participants cannot access—participants should not be left without support and could be offered alternative interventions (not requiring mobile data access) following the completion of the study.

When planning to recruit school-aged individuals to real-time monitoring studies, extra consideration should be given to data collection within the school context. For instance, schools may prohibit access to devices during the school day. van Roekel and colleagues [94] provide useful recommendations for working in school contexts, such as ensuring that there is a strong alliance with schools, teachers, and parents by using participation cards so that students are allowed to use their phones when prompted, and making sure that schools also benefit from the research. Having a specific person who is the “face” of the study within the school can also be useful. For a detailed discussion of the challenges of conducting NSSI research generally within schools, see [95].

### Responsibility 2: Informed Consent and Participant Briefing

Given that real-time monitoring research takes place in daily life without the researcher being present, additional consideration of the informed consent process and participant briefing is needed [96]. Information regarding the study’s often intensive nature, such as the study’s time course, the number of surveys per day, and the periods during which participants can expect prompts, should be made clear to potential participants before study enrollment. Given that participant compliance rates in real-time monitoring research can vary [97], participant briefing should cover whether financial compensation or other benefits of research participation are compliance-dependent and, if so, how many reminders will be sent. When studying NSSI, in particular, it is paramount that participants are informed about the safety procedures (especially when this involves human-led intervention contingent upon a survey response) and the potential consequences of these safety procedures (eg, when will the duty of care override the confidentiality principle and who will then be informed). The informed consent process should also clarify whether additional data will be passively collected (eg, location coordinates, incoming and outgoing SMS messages and calls, app usage statistics, accelerometer data) and make participants aware of the detailed level of data that can be collected *without* their active engagement. Poor digital literacy may threaten adequate informed consent [98], especially for passively collected data. Jacobson and colleagues [96] provide several valuable suggestions to ensure that participants have a complete and detailed understanding of the study, such as highlighting essential information, using comprehension quizzes, and preventing participants from scrolling through the informed consent without reading it (when provided online). Considering the extensive amount of information participants receive, it could be worthwhile to request consent for each part of the study separately (eg, data collection schedule, intervention component, safety protocols/plan). Providing real-world examples utilizing interactive videos or apps that can read information aloud could also be used to facilitate comprehension and mitigate the risk of poor digital literacy.

When working with minors, both the young person’s *assent* and informed *consent* from their parent or caregiver will typically be required. However, as a highly stigmatized behavior, NSSI is often hidden from others [99]. Although disclosure to parents and caregivers can facilitate help-seeking and improve coping, it can also negatively impact the parent-child relationship and the wider family system [66,100] and lead the young person to worry about the involvement of parents or caregivers [101]. Although parents will often be informed when recruiting young people within a clinical setting, we recommend explaining the study’s purpose in general (eg, to study interactions, emotions, thoughts, and behavior in daily life) instead of using NSSI-specific terms to avoid forced disclosures. This framing method also means that individuals may avoid reflecting upon their participation through a disease perspective or NSSI-labeled identity [102,103]

### Responsibility 3: Participant Safety and Risk Monitoring

In real-time monitoring studies of suicidal thoughts and behaviors, ethical considerations regarding participant safety are, justifiably, a recurring concern [96,104]. In contrast, very few NSSI real-time monitoring studies report procedures for safeguarding and supporting participants during the study [101]. This may be because high suicide risk is sometimes an exclusion criterion for participation [33,105,106], and participant safety procedures are generally to safeguard participants at high or imminent risk of making a suicide attempt [27,38]. Real-time monitoring studies tread a fine line between research and intervention and there must be a “goodness of fit” between a study’s objectives and the design of the safety procedures [101]. For example, in a study of NSSI behavior, contacting participants every time they report engaging in NSSI would defeat the study’s purpose and may even discourage participants from reporting NSSI during the study period [101]. If a participant scores highly on a momentary measure of suicidal intent, contacting the participant may be appropriate and would not compromise the study’s goal of assessing NSSI. A critical ethical issue underlying participant safety procedures is that even though an increase in suicidal intent is unlikely to be caused by study participation [107,108], the individual’s status as a participant in a real-time monitoring study creates an opportunity for intervention that would otherwise not exist. Therefore, we recommend assessing suicidal intent in real-time monitoring research on NSSI thoughts and behaviors and advise against the exclusion of people at risk of suicide.

The first consensus statement on ethical and safety procedures for real-time monitoring studies with individuals at risk of suicide has emerged recently [109]. The recommendations include collecting contact information for participants and a close contact, completing a safety plan at study enrollment, monitoring responses at least once per day, and in the event of a participant being at imminent risk of suicide, for a researcher to contact them. Interestingly, our experiences have been somewhat different, with clinicians expressing concern that a researcher may be the “first responder” to a suicidal crisis. In this regard, contacting the participant’s clinician may be better. However, no consensus was reached regarding whether the researcher should contact a participant’s clinician in the event of high or imminent suicide risk [109]. The logistical challenges of actively monitoring participants’ responses and potentially intervening should not be underestimated, especially for large studies where multiple participants may require intervention simultaneously. Ensuring that adequate staffing and resources are available to carry out the study’s safety procedure is essential. In the interests of transparency and to evaluate safety procedures in real-time monitoring studies of NSSI, we recommend reporting details regarding participant safety protocols as standard. Moreover, qualitative research should substantively investigate participants’ and clinicians’ preferences for safety procedures concerning NSSI outcomes.

### Responsibility 4: Measurement Reactivity

A particular concern in all NSSI and suicide research is that asking individuals questions about their self-harm–related thoughts and behaviors may inadvertently increase the likelihood of the individual thinking about or engaging in self-harm. However, evidence suggests that this is not the case [110,111], prospectively across young adult [112], adult, and adolescent samples [113,114], and when using various NSSI-related stimuli (eg, images, words) [113]. In contrast, findings indicate that participants find their participation in research on NSSI and suicidal behavior to be beneficial [110,112,114]. Although it appears that asking people about NSSI and suicide at a single time point has no impact on self-harm–related thoughts and behaviors, real-time monitoring requires repeated questioning on these topics. To date, no research has tested the potential iatrogenic effects of real-time monitoring research specifically for NSSI, but evidence from the suicide literature is promising. Early work by Husky and colleagues [107] used real-time monitoring to assess depression, mood, and thoughts of suicide and self-harm 5 times a day for 1 week in 4 samples: people with a recent suicide attempt, people with a past suicide attempt, people with mood disorders but no suicidal behavior, and healthy controls. In this study, there was no reactivity to the repeated questioning about self-harming thoughts across any of the 4 groups. Law and colleagues [105] demonstrated similar outcomes with a longitudinal design assessing 248 adults (30% of whom reported a borderline personality disorder diagnosis, which confers additional suicide risk). In this study, the authors found no increase in suicidal thoughts and behaviors during the initial 2-week data collection phase nor at the 6-month follow-up, including for people with a borderline personality disorder diagnosis. Recently, Coppersmith and colleagues [108] found no association between the frequency of asking about suicidal ideation and intent in a real-time monitoring study and the severity of suicidal thoughts over time. They also observed no change in survey responses when ideation was severe, where a decrease might be expected if participants were reactive to questioning. Currently, the evidence suggests no iatrogenic effects of repeated questioning about suicide and suicide-related behaviors. Further research is required to confirm whether this pattern also holds true for NSSI and is robust across different subgroups.

### Responsibility 5: Balancing Scientific Accuracy Against Participant Burden and Ensuring the Research Is Feasible, Transparent, and Safe

An important responsibility when designing a real-time monitoring study is the selection of an appropriate sampling design (ie, fixed, interval, [semi-] random, event-based, mixed), sampling density (ie, number of assessments per day), sampling duration (ie, number of days/weeks), and sample size [45,94]. It is crucial that the selected sample shows sufficient variability in the outcomes of interest to allow investigation of the research question in daily life [45]. For example, when the aim is to clarify the transition from NSSI thoughts to behavior, base rates of thoughts and behavior must be high enough during the real-time monitoring period. To ensure this is the case, researchers need to consider the inclusion criteria carefully (eg, by including individuals with more than 5 acts of NSSI behavior in the 2 weeks before study onboarding). The selected protocol should allow answering the prespecified research question(s) without unnecessarily burdening the participants. Balancing these needs may mean that protocols are not interchangeable across studies—there is no “one-size-fits-all” protocol. Researchers should be cautious about, for example, adopting sampling densities used in previous studies, as these may not be suitable for addressing different research questions. In some cases, it will be perfectly justifiable—even essential—to ask participants to complete a more intense or extended real-time monitoring protocol. However, to ensure divergence between protocols across studies is not arbitrary, researchers must justify their protocol [115] and communicate expectations to potential participants during study onboarding.

Compared with other research methodologies, real-time monitoring studies involve a higher workload for and burden on participants. Therefore, we recommend piloting the feasibility and acceptability of real-time monitoring protocols by using a quality improvement procedure, where protocols are first tested extensively by members of the research team and then iteratively with a selected group of participants. In our experience, this approach requires more time and planning but safeguards participants’ (and researchers’) investment by allowing the protocol to be modified and optimized, if needed, in response to qualitative and quantitative feedback (eg, the time it takes to complete the questionnaire). Emerging evidence suggests that the questionnaire length, rather than sampling frequency, is associated with increased participant burden and reduced data quality [116]. Although these findings are hopeful for researchers wanting to use more intense protocols, they also underscore the necessity for careful item conceptualization and selection. To avoid a methodological “Wild West,” we advise researchers to make their items publicly available (see the Experience Sampling Method Item Repository) [117] and report (where possible) the multilevel reliability and validity of operationalized NSSI outcomes. Against the backdrop of the replication crisis, preregistration and data sharing (open data) are also increasingly used to increase reproducibility and avoid wasting public resources [118]. These considerations are especially relevant considering the high demands of a real-time monitoring study on participants and researchers. For a tutorial and template for the preregistration of real-time monitoring studies, see Kirtley and colleagues [119]. Finally, there is consensus among researchers and clinicians that real-time monitoring platforms should be secure and compliant with the relevant privacy laws [109]. In some cases, data collected with real-time monitoring apps may be stored in another country and thus subject to different privacy legislation from the researcher’s home country. Researchers should thus be well-informed before commencing a real-time monitoring study and when unsure, should contact their institution’s research governance or data management department for clarification.

### Responsibility 6: Compensation and Recognition of Participant Engagement

Consideration should be given to distributive justice principles, such that participants benefit from their engagement in the study. Prior work found that the majority of young people who take part in traditional survey research investigating sensitive topics, such as NSSI, report that their participation allowed them the opportunity to develop greater self-awareness and had altruistic value [114]. However, real-time monitoring studies tend to require more time and effort from participants. To give some initial insight into the experience and expectations of participants who self-injure, we present additional results from a recent real-time monitoring study in which emerging adults were prompted every 90 minutes during waking hours for 12 days [35].

Following the monitoring period (n=29), approximately 4 of 5 participants indicated increased self-awareness of feelings and thoughts (Table 1). About half considered the protocol demands to be tiring, but most participants also described their participation as positive. Notably, all participants reported being interested in receiving information on the key findings of the study when it concluded. Although not a primary reason for participation for most participants, they also expected feedback on their own data (21/29, 72%) and a monetary incentive (23/29, 79%). Participants considered €60 (€1=US $1.15) to be a fair compensation for the study protocol’s demands (8 beeps/day for 12 days, 96 assessments in total, median compliance 79.2%, IQR 70.3%-91.7%), when they also received feedback about the overall findings and their own data. In the absence of feedback on their data, participants expected higher financial compensation for their investment. In this study, participants were reimbursed according to a structured financial scheme to encourage participation. Rather than paying a fixed amount per completed survey, a structured incentive scheme has the advantage that it allows participants to miss some surveys without direct financial consequences. For some groups (eg, adolescents, people with low socioeconomic status), avoiding direct financial consequences of missed surveys may be particularly important to prevent some participants from changing their daily routines to respond to each survey.

These findings are consistent with those of the traditional survey research [114] and provide the first indication that real-time monitoring offers self-awareness opportunities for participants. Further, it highlights to researchers that they should actively recognize participants with lived experience of NSSI as valued partners in the research by also giving informational support. Although the majority experienced participation as positive, future work should clarify why this may not be the case for everyone so that resources can be provided to those for whom participation may have increased burden and discomfort. In this respect, it is advisable to organize a debriefing session that allows participants to share their experiences. Moreover, future work could explore the perceived meaningfulness of several strategies, such as the tailoring of sampling schedules and including a temporary “suspend” button to offer participants flexibility and greater control over notifications [115], the randomization of items to reduce response fatigue (called planned missing data designs) [120,121], and allowing for catch-up days so that participants can reach the desired level of compliance [94].

**Table 1:** Experience and expectation of individuals with lived experience of nonsuicidal self-injury (n=29).^a^

Experiences and expectations		Disagree to disagree completely	Agree to agree completely
**Subjective experience, n (%)**			
	By completing the questions in everyday life, I became more aware of how I felt	4 (14)	23 (79)
	By completing the questions in everyday life, I became more aware of my thoughts	3 (10)	21 (72)
	Participating in the smartphone study was tiring	7 (24)	14 (48)
	I would describe my participation as a positive experience	3 (10)	19 (66)
**The overall importance of receiving study findings, personal feedback on own data, and financial compensation, n (%)**			
	I am interested in the overall results of the study	0 (0)	29 (100)
	Receiving personal feedback is an important reason for me to participate	8 (28)	13 (45)
	Receiving financial compensation is an important reason for me to participate	4 (14)	12 (41)
**The relative importance of general and personal feedback and financial compensation, n (%)**			
	If I receive feedback on the overall results, financial compensation is not necessary	23 (79)	1 (3)
	If I receive feedback on the overall results, personal feedback is not necessary	21 (72)	5 (17)
	If I receive financial compensation, personal feedback is not necessary	23 (79)	2 (7)
	If I receive personal feedback, financial compensation is not necessary	18 (62)	5 (17)
**Expected financial compensation (Euros), €1=US $1.15, median (IQR)**			
	What amount do you consider fair as compensation when you also receive general and personal feedback?	N/A^b^	60 (45.0-75.5)
	What amount do you consider fair as compensation when you receive general but not personal feedback?	N/A	70 (55.0-81.5)

^a^Unpublished data of 29 young adults with lived experience following participation in a 12-day real-time monitoring protocol with 96 semirandom longitudinal assessments (8/day, median compliance 79.2%; IQR 70.3%-91.7%; 29/30, 97% retention [35]). The response category “neutral” is not shown in the table.

^b^N/A: not applicable.

### Responsibility 7: Researcher Well-being and Training

Finally, we want to draw explicit attention to researcher well-being and training considerations. Real-time monitoring data are quite literally an individual’s real-life experiences, occurring in real-time, which may lead researchers to feel especially close to participants and their experiences. Although studies have explored participants’ experiences of taking part in research on self-harm and suicide more broadly [122], as well as in real-time monitoring studies [123,124], to our knowledge, no studies have investigated researchers’ experiences of conducting real-time monitoring studies of NSSI or suicidal behaviors. However, the stakes are high when the research focus is on self-injurious behavior; monitoring participants’ responses for signs of imminent suicidal crisis lays great responsibility on researchers’ shoulders. Where safety protocols involve routine telephone check-ins, a missed check-in may cause the researcher to fear for the participant’s safety even though there may be an innocuous explanation (eg, the participant was driving or in the shower). Researchers may also begin to feel a high level of responsibility for participants not engaging in NSSI or attempting suicide (ie, feeling they are keeping the participant alive or safe) [125,126]. Studies that employ real-time alerts when participants indicate escalating suicidal intent may lead researchers to become hypervigilant and worry that intervention may be required at any moment. Such warnings may come outside of working hours, thereby increasing work-life balance challenges. For the researcher, all of these situations underscore a lack of controllability, which may prove highly stressful, especially when experienced over a sustained period.

Good quality training in working with individuals who engage in NSSI and suicidal behaviors is essential when conducting real-time monitoring research. Much research is carried out by doctoral students, trainees, and research assistants, who may be less experienced in collecting sensitive data and managing the accompanying emotional labor [126]. Researchers often work alone and lack a good support network [127]. Therefore, training should cover supporting a participant during an acute suicidal crisis as well as less overtly “intense” situations (eg, where researchers are coding open-text responses about participants’ reasons for engaging in NSSI). A lone researcher should never carry out safety protocols involving real-time risk-monitoring and intervention and responsibility should be shared among a team, whereby multiple researchers are “on-call” for a specified period. Although planning is essential, some researcher well-being challenges may only become known once the study is underway [126]. Continued supervision and mentoring of researchers (of all career stages) are crucial to ensuring researcher well-being. This may involve regular debriefings with supervisors or colleagues or via external, independent counseling support. Qualitative studies with researchers who work on sensitive topics also highlight the importance of self-care and actively engaging in positive, non–work-related activities as valuable buffers against emotional distress [126,127].

## Summary of Responsibilities

**Figure 1:**
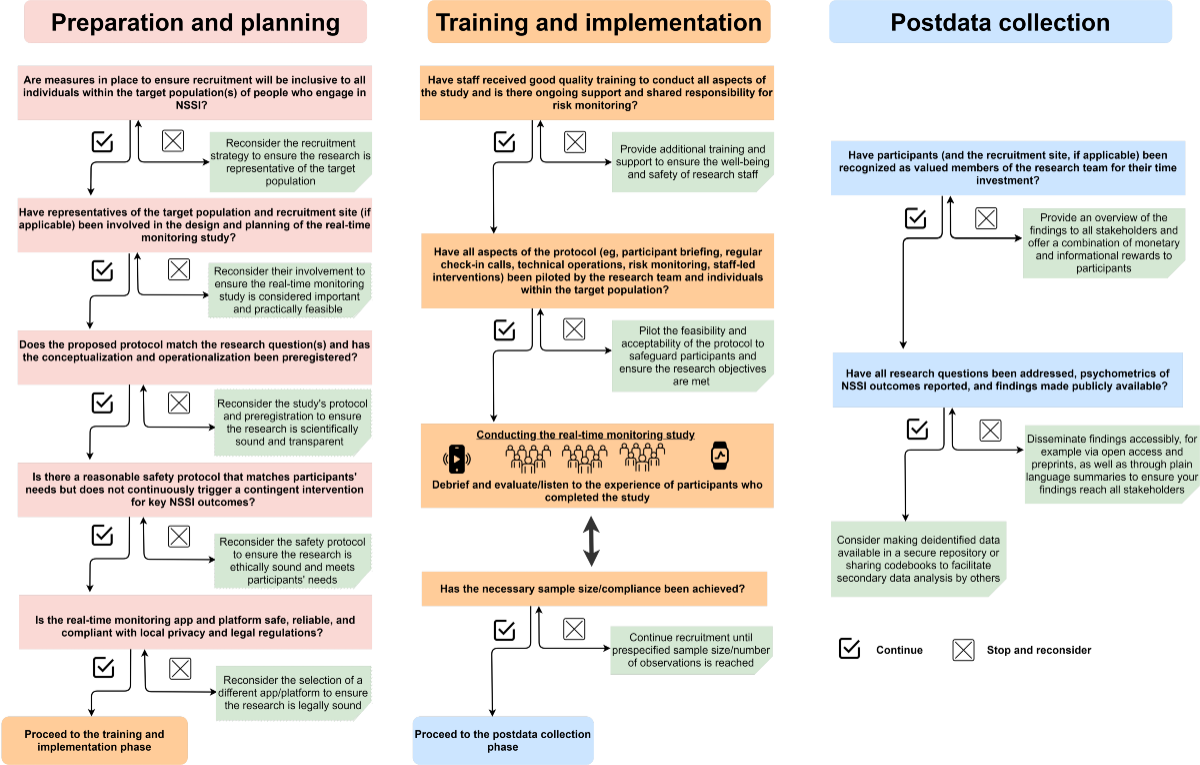
Flowchart of the critical considerations when opening the black box of daily life in nonsuicidal self-injury research. NSSI: nonsuicidal self-injury.

Real-time monitoring technologies give researchers the practical tools to investigate NSSI thoughts, urges, and behavior as they occur, without the need to be physically present. Although this provides immense opportunities for NSSI research, real-time monitoring research is not without challenges regarding recruitment, study enrolment and planning, and participant engagement. Focusing on NSSI also creates great responsibility for privacy and data security, participant safety and risk-monitoring, and researcher well-being and training. To guide researchers who want to study the experiences of individuals who engage in NSSI, we summarize the responsibilities and ways of overcoming challenges in a functional flowchart (Figure 1).

## Where Do We Go From Here?

Since Nock et al’s seminal study in 2009 [27], which demonstrated the feasibility of studying NSSI in adolescents’ everyday life, researchers now have a toolbox full of smartphone apps and wearable technology that can readily capture real-time experiences of people who engage in NSSI in the real-world context. These advances produce a rapidly growing literature that could positively shape the field’s future trajectory by facilitating a radical shift of focus from the group to the individual, from the research lab and clinic to the everyday life environment, and from traditional generalized treatment to person-centered prevention and intervention. This paper sets an ambitious agenda for new research pathways to realize such a shift as we move into the next decade of NSSI research. We also offered critical perspectives on the inevitable ethical and practical challenges that come with these research pathways. In this respect, opening the black box of daily life in NSSI research is truly a double-edged sword that requires responsibility and leads to new questions. Few studies to date have specifically considered ethical issues within real-time monitoring studies of NSSI. However, these questions regarding ethical practices must be addressed with substantive empirical research rather than being based upon the precedent of “if it ain't broke, don't fix it.” In particular, research co-design and qualitative studies to capture rich information on participants’ experiences of taking part in real-time monitoring research on NSSI offer promise. Real-time monitoring research on NSSI will advance better and more rapidly when all stakeholders’ interests (ie, individuals with lived experience, their families, researchers, and clinicians) are considered. Only by considering both the opportunities and the challenges will we be able to use real-time monitoring techniques to their full potential.

## Abbreviations

EMI: ecological momentary intervention
JITAI: just-in-time adaptive intervention
NSSI: nonsuicidal self-injury
